# Advancing Cancer Therapy with Quantum Dots and Other Nanostructures: A Review of Drug Delivery Innovations, Applications, and Challenges

**DOI:** 10.3390/cancers17050878

**Published:** 2025-03-04

**Authors:** Ashutosh Pareek, Deepanjali Kumar, Aaushi Pareek, Madan Mohan Gupta

**Affiliations:** 1 Department of Pharmacy, Banasthali Vidyapith, Banasthali 304022, Indiaaaushipareek@banasthali.in (A.P.); 2 School of Pharmacy, Faculty of Medical Sciences, The University of the West Indies, St. Augustine, Trinidad and Tobago; 3 Nims Institute of Pharmacy, Nims University Rajasthan, Jaipur 303121, India

**Keywords:** quantum dots, breast cancer, bioimaging, drug delivery, fluorescence

## Abstract

Nanotechnology, particularly quantum dots (QDs), has ushered in a transformative era in the pharmaceutical and medical industries, offering notable opportunities for nanoscale advancements. These nanoscale particles, known for their exceptional optical properties and quantum confinement, have emerged as indispensable tools in cancer drug delivery and bioimaging. This review delves into various drug conjugation techniques with QDs, including covalent linking, non-covalent conjugation, click chemistry, disulfide linkage, and pH-sensitive linkage. Each method provides distinct advantages, such as enhanced stability, reversibility, specificity, and controlled drug release. Moreover, QDs have demonstrated significant promise in oncology by efficiently delivering drugs to cancerous tissues while minimising systemic toxicity. Investigations into their applications in different cancers, such as blood, brain, cervical, breast cancers, etc., reveal their efficacy in targeted drug delivery, real-time imaging, and improved therapeutic outcomes. However, challenges such as potential toxicity, stability, pharmacokinetics, and targeting specificity must be addressed to fully harness the benefits of QDs in cancer therapy. Future research should focus on developing biocompatible QDs, optimising conjugation techniques, and elucidating their safety profiles and long-term effects in biological systems. Overall, QDs represent a promising frontier in cancer treatment, offering multifaceted capabilities that hold the potential for enhanced therapeutic outcomes and reduced side effects across various cancers.

## Simple Summary

This paper discusses the use of quantum dots as drug delivery nanocarriers, especially for cancer. With their small size and unique optical properties, they contribute significantly to the therapeutic efficacy of chemotherapeutic agents and live tracking of the same while minimising off-target effects. Different conjugation techniques of quantum dots and drug molecules explain the adaptability of quantum dots, tailored to their needs. The current state of the research on quantum dots in various cancers, such as breast, liver, lung, cervical, pancreatic, etc., is also mentioned, focusing on the benefits offered by quantum dots as nanocarriers. However, the challenges associated with them impede their way towards clinical translation, but future research perspectives focus on developing biocompatibility and addressing clinical limitations.

## 1. Introduction

Nanotechnology has revolutionised the pharmaceutical and medical industries with its transformative potential, paving the way to early diagnosis and drug delivery [1,2]. At the forefront of this nanotechnological revolution are quantum dots (QDs), nanoscale zero-dimensional crystals [3] known for their various optical, spectral, magnetic, and electrochemical properties [4]. Among the various nanomaterials, QDs have shown substantial attention as an indispensable tool in cancer drug delivery and bioimaging [5].

QDs vary in the 2–10 nm size range [6,7,8,9], but they may also range up to 100 nm in size depending on the coating [10]. QDs emit light across a spectrum of wavelengths, depending on their particle size [11]. The phenomenon, driven by quantum confinement effects [8,12], makes QDs particularly suitable for bioimaging [13] and tracking drug delivery [14].

**Figure 1:**
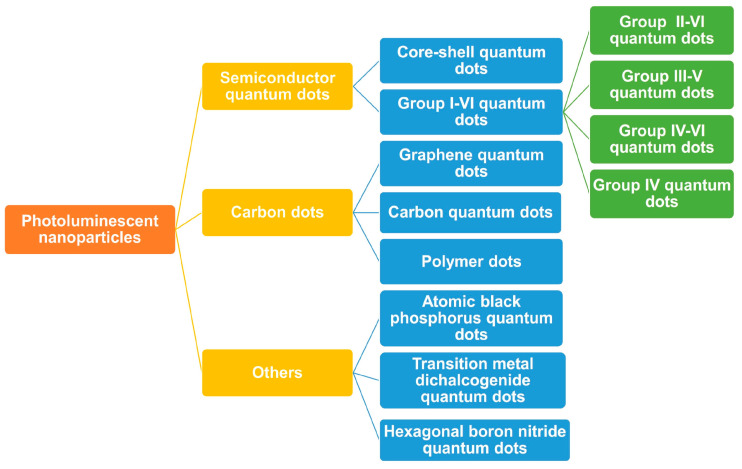
Classification of quantum dots based on material composition.

QDs are categorised by composition, consisting of either single-element materials like silicon and germanium or compound semiconductors, such as CdSe, CdTe, lead sulfide (PbS), lead selenide (PbSe), and carbon materials. Photoluminescent nanoparticles are classified into different types: semiconductor QDs (SQDs), carbon dots (CDs), and others [15]. The detailed classification is given in Figure 1.

SQDs are made up of semiconductor materials that exhibit photoemission with good photostability. This emission is dependent on the size of the QDs and their chemical composition. Smaller QDs (2–3 nm diameter) have a larger bandgap, leading to emission at shorter wavelengths in the blue region (UV), whereas larger QDs (5–6 nm) possess a smaller bandgap, consequently emitting light at longer wavelengths in the red region (NIR). The bandgap also depends upon the elements composing QDs as the bandgap energy lowers on progressing from binary group I to VI in the periodic table [16]. Further, they are categorised according to the elements they constitute in the periodic table [17]. They are typically composed of an inorganic crystalline core—such as cadmium selenide (CdSe) or cadmium telluride (CdTe)—and encased in an inert zinc sulphide shell [18]. These materials enable QDs to emit visible or near-infrared (NIR) range-triggered fluorescence and give size-tunable optical properties [19,20]. Core-shell QDs provide better quantum yields and photostability by shielding the core from oxidation and degradation, leading to brighter, more stable fluorescence [21]. QDs are also categorised based on the groups in the periodic table, including elements from different groups. For example, Groups I-VI include Cu and Ag_2_ coupled with sulphur(S), Se, and Te, making them useful for easy bioconjugation and thus suitable for targeted delivery and bioimaging [22]. Groups II-VI SQDs primarily include heavy metals like Cd and Zn coupled with S, Se, and Te [23], which are employed for electrochemical immunosensing in cancers [24]. Groups III-V SQDs, including indium (In) and gallium (Ga) coupled with phosphide, nitride and arsenic, are known for their low toxicity and high fluorescence [25]. Groups IV-VI SQDs constitute elements like lead (Pb) with S, Se, and Te, and their optical properties are dependent on their size [26]. They respond sensitively to NIR light [27]. Group IV SQDs include carbon (C), germanium (Ge), and silicon (Si). They can be useful for deep-tissue penetration, bioimaging, and drug delivery due to their ability to produce light emission in infrared regions [28].

The toxicity concerns associated with SQDs have limited their use to bioimaging, mostly with surface modifications, which has led to a shift towards CQDs and GQDs in cancer research. For example, CdSe/ZnSe/ZnS QDs were developed for the detection of human ovarian cancer [29]. Surface-modified CdSe/CdS/ZnS QDs with transferrin and anti-claudin-4 were used for the imaging of pancreatic cancer cells [30]. Similarly, cysteine-conjugated CdSe/CdS QDs were used as an optical biosensor for the detection of skin cancer [31].

CDs are composed of carbon materials with sizes less than 10 nm [32]. Broadly, they are carbon nanoparticles [33] and can be classified into CQDs, polymer dots (PDs), and graphene QDs (GQDs). CQDs are low molecular weight photoluminescent QDs exhibiting quantum confinement [34]. Various functional groups are present on the surface of CQDs, namely COOH, NH_2_, and OH, offering sites for surface conjugation [34]. They have low cytotoxicity and high biocompatibility with fluorescence emission, collectively making them suitable for the bioimaging of cells and tissues and drug delivery. The outstanding biocompatibility and minimal toxicity of CQDs enable their effective uptake into cellular structures [35]. Quantum confinement in CQDs leads to the separation of energy levels, resulting in a size-dependent bandgap. When the nanomaterial absorbs a photon, an electron transitions from the valence band to the conduction band, forming an electron-hole pair, an exciton. Upon exciton recombination, the excess energy is emitted as fluorescence [36]. CDs are structures with at least one dimension smaller than 10 nm and are notable for their photoluminescent properties, with oxygen/nitrogen-containing functional groups [37]. Their excitation-wavelength-dependent photoluminescence arises from the quantum confinement of surface trap states on individual CD surfaces, which has been researched and proven by Bhattacharya et al. [38]. GQDs are zero-dimensional QDs composed of multiple graphite thin sheets [34], with sizes ranging up to 20 nm and with oxygen and hydrogen as functional groups. Their unique carbon structure and specialised surface modifications enhance biocompatibility, reduce toxicity, and promote biodegradability, making them more suitable for biomedical applications [39]. The photoluminescent properties are determined by both the graphene core and the chemical groups in its surrounding environment. Also, they exhibit a broader energy band, which is responsible for the photoluminescence of most GQDs, primarily in the blue to green spectrum region [40]. The photoluminescence of these materials ensures accurate and controlled monitoring of the therapeutic agents throughout the transport process. They frequently exhibit photoluminescence, which also depends on the excitation wavelength [41]. This property makes them suitable for photothermal therapy (PTT) and photodynamic therapy (PDT) [41]. Properties like pH stability, water solubility, photo stability, and corrosion resistance also favour their use in PDT [42]. GQDs, upon absorbing visible radiation, produce reactive oxygen species (ROS) that lead to cytotoxic effects in cancer cells [43].

Furthermore, QD nanocarriers have a high surface area-to-volume ratio, enabling multifunctionality through the conjugation of drug molecules, targeting ligands, and other functional coatings [44]. Additionally, certain QDs can bypass the blood–brain barrier (BBB), making them an effective tool for delivering drugs directly to tumour sites [45,46]. For example, CdSe/ZnS QDs conjugated with captopril crossed the BBB through transcytosis-mediated patterns and ultimately reached the brain parenchyma [47]. CQDs without targeting ligands have been proven to cross the BBB [48], and GQDs could also effectively penetrate the BBB in vivo [49]. QDs can also exploit the leaky vasculature of the tumour blood vessels to passively leak out of the bloodstream and accumulate within the tumour tissue [50,51,52]. QDs with specific ligands allow for targeted delivery to particular cells or tissues, enhancing drug efficacy and minimising side effects [53]. Quantum dots (QDs) are surface coated to augment their solubility and stability by hydrophilic ligands adorned with functional groups, such as COOH, OH, and SH, which bestow QDs with water solubility. This layer of coating shields the outermost QD layer from degradations. They can also be surface coated with wider-band-gap semiconductor material, resulting in increased optical efficiency [54]. Their nanoscale size facilitates penetration through biological barriers, enabling access to deep tissues, making QDs a compelling subject of research in drug delivery [55].

Furthermore, the prolonged circulation of QDs in the bloodstream enables their passive accumulation at tumour sites through the leaky tumour vasculature via the enhanced permeability and retention (EPR) effect [56]. The nanoscale size of QDs allows them to be readily taken up into cells through endocytic pathways [57]. QDs fall below the size limit of cellular entry, i.e., 2–10 nm through endocytosis, which is typically around 200 nm [58]. Despite their small size, QDs have demonstrated an exceptional capacity to bypass degradative lysosomal pathways after endocytic uptake, enabling prolonged intracellular retention and enhanced therapeutic efficacy [58,59]. They remain sequestered in the endolysosomal compartments for an extended period. This retention facilitates efficient cytosolic drug delivery and targeted therapeutic effects at intracellular sites of action. Leveraging these properties, researchers have developed various drug conjugation strategies to optimise intracellular delivery using QDs [60].

In recent years, the convergence of nanotechnology and oncology has driven groundbreaking advancements in cancer diagnosis and therapy, with QDs emerging as a powerful tool in the fight against cancer.

This review aims to highlight the research done so far on QDs as drug delivery nanocarriers against a variety of cancers, along with the challenges, and explore the future prospects following this.

## 2. Approaches to Drug Conjugation onto QDs

A variety of intricate approaches are utilised to conjugate drugs with QDs, each offering unique benefits. These methods enable precise drug delivery, sustained release, and improved therapeutic outcomes. Below are the key strategies employed.

### 2.1. Covalent Linking

**Figure 2:**
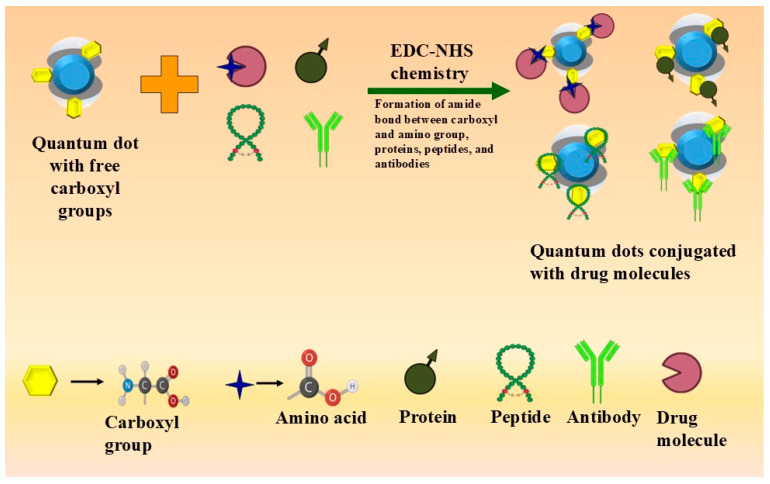
Schematic illustration of covalent conjugation using EDC-NHS chemistry, which enables the formation of bonds with free amino groups, proteins, peptides, and antibodies.

Covalent linking is a reliable technique for binding drug molecules onto QDs, ensuring stable and durable linkages. This approach enhances the therapeutic effectiveness of drugs by facilitating sustained and targeted drug release [61]. Covalent bonding typically involves binding the free amino and carboxylic groups of QDs to corresponding groups on drugs or biomolecules [62]. There are various mechanisms for covalent linking, including the following:

–COOH-containing QDs, which provide easy conjugation with free amino groups, carbohydrate hydroxyl groups [62], proteins, peptides, and antibodies [63].
- EDC-NHS (N-hydroxy succinimide) chemistry: Employing 1-ethyl-3-dimethylamino propyl carbodiimide hydrochloride (EDC) as a cross-linker to form an amide bond between the carboxyl group of QDs and the amine group of the biomolecule [64]. This method is commonly used for targeting cells through receptor-mediated endocytosis and folate receptor-mediated delivery [65]. Figure 2 depicts the covalent conjugation.
- Conjugation of biomolecules with –NH2-containing QDs is a widely used technique involving the thiol group on the molecules [58]. Reagents like Sulpho-SMCC [Sulfosuccinimidyl-4-(N-maleimidomethyl) cyclohexane-1-carboxylate] are frequently employed [66]. QDs with amino groups are used for conjugation with peptides, proteins, receptors, and antibodies [67].
- Thiol-containing QDs can bind to sulphur-containing compounds, such as amino acids, through disulfide bonds, thereby enhancing the solubility of hydrophobic QDs [64]. Similarly, epoxide-containing QDs react with amine, thiol, or hydroxyl molecules, forming stable secondary amine, thioether, or ether bonds [64].

Iannazzo et al. [68] conjugated BFG (methyl3,3-dimethyl-2-(3-methyl-2,3-dihydrobenzofuran-2-yl) butanoate) covalently onto the surface of GQDs using a cleavable PEG linker designed to be selectively activated within human cells.

### 2.2. Non-Covalent Conjugation

Non-covalent conjugation is an attractive technique due to its versatility and reversible nature. It allows for the binding of various drugs onto QD surfaces without altering their chemical structure, preserving the drug’s bioactivity and structural integrity. Non-covalent interactions include electrostatic forces, hydrophobic interactions, and van der Waals forces [69].

For example, prior research investigated the in vitro potential of supramolecular hybrid CDs in conjugation with DOX in the treatment of cancer. DOX was conjugated non-covalently onto QDs through electrostatic interactions along with π–π stacking. The non-covalent conjugation proved to be advantageous in several respects, primarily by preserving the activity of DOX. Unlike covalent conjugation, which involves forming chemical bonds between the drug and the carrier, non-covalent interactions allowed DOX to remain in its native form without undergoing chemical modifications. This preservation of the drug’s structure and properties ensured that DOX retained its therapeutic activity. The study also highlighted pH-dependent drug release, triggered in the acidic tumour microenvironment, ensuring localised drug release [70].

### 2.3. Click Chemistry

Click chemistry involves highly selective and efficient reactions between specific functional groups of QDs and drugs, offering precise attachment without interfering with other components. Techniques such as copper-catalysed azide-alkyne cycloaddition (CuAAC) and strain-promoted azide-alkyne cycloaddition (SPAAC) are commonly used [71].

In the context of drug delivery onto QDs, the bio-orthogonal nature of click chemistry is exploited to achieve precise control over drug delivery, which ultimately refines the therapeutic potential of the drug delivery system. Bio-orthogonality is a concept that ensures the feasibility of a reaction under physiological conditions such as pH and temperature [72]. In addition, click chemistry is adaptable to a diverse array of drug compounds and quantum dot surfaces, offering flexibility in drug linkage techniques.

To shed light on conjugation carried out using this technique, Chen et al. [73] functionalised the outer layer of zinc sulphide (CdSe/ZnS) QDs by attaching histone deacetylase inhibitors (HDACi) through click chemistry (azide-alkyne cycloaddition). The conjugation formed a hydrophilic nanohybrid. Most importantly, the functionalisation process was carried out under mild conditions to reduce any damage caused to the biological components. As a result, the biological activity of HDACi and QDs was preserved. The resultant nanohybrid exhibited improved biological effects, such as significant suppression of lung cancer cell growth and elevated acetylation levels of histone and nonhistone proteins. This demonstrates the efficiency of click chemistry-based functionalisation in generating the intended biological activity, emphasising the benefit of adopting click chemistry in this context.

### 2.4. Disulphide Linkage

Disulfide bonds are utilised to conjugate drugs to QD surfaces, enabling controlled release in the intracellular environment. This approach ensures the stability of conjugated drug molecules in oxidising environments and targeted drug delivery to cancer sites [43]. Sangtani et al. [74] investigated DOX-peptide complexes conjugated to QDs via ester, disulfide, and hydrazone linkages. Among these, disulfide linkages provided the highest stability and least toxicity, resulting in sustained drug release in endosomal compartments.

### 2.5. pH-Sensitive Linkage

Another emerging technique is the usage of pH-sensitive linkers, through which drugs can be conjugated onto QDs and explicitly released in the acidic tumour microenvironment, elevating the localised therapeutic effect while cutting down systemic exposure [75].

**Figure 3:**
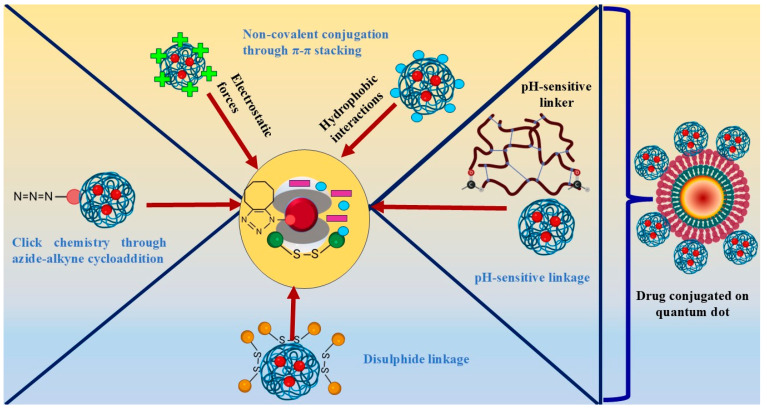
Schematic representation of different drug conjugation strategies with QDs, including non-covalent interactions, click chemistry, disulfide linkages, and pH-sensitive linkers.

In a study by Chen et al. [45], the role of a pH-sensitive linker was investigated in conjugating DOX to fluorescent CQDs to boost the specificity and effectiveness of the drug delivery system. The pH-sensitive linker utilised in the research was a hydrophobic linear reducible-responsive linker with two terminal aldehyde groups. This linker was engineered to react to pH variations in the acidic tumour microenvironment to trigger drug release in a controlled manner, thereby causing little to no damage to the healthy surrounding cells. The pH-sensitive linkage resulted in the stability of the drug within the CQDs until it reached the tumour site. This mechanism effectively prevented any untimely drug release and degradation, thus ensuring the optimal therapeutic efficacy of the drug. The conjugated drug delivery system outperformed free drug formulations in effectively suppressing tumour growth, emphasising the benefits of pH-sensitive drug conjugation. A schematic illustration of non-covalent conjugation, click chemistry, disulphide linkage, and pH-sensitive linkage is depicted in Figure 3.

## 3. QDs in Oncology

QDs have shown immense potential in oncology because of their unique properties, which make them ideal for targeted drug delivery and imaging in cancer treatment [76]. QDs are valuable assets in diagnostics [3], recognised for their fluorescence intensity. They facilitate size-dependent light emission and can generate a range of fluorescent colours from a single excitation source, enhancing their utility in diagnostic applications. Such multicolour QDs can be utilised as probes to analyse multiple molecular targets simultaneously. QDs exhibit superior detection sensitivity compared to conventional organic dyes, establishing them as highly effective fluorophores that significantly improve cellular imaging. They show strongly exceptional resistance to photobleaching, unlike organic fluorophores [15,77], and can be thus used for long bioimaging therapies without signal degradation [78]. They possess a higher quantum yield than organic dyes and fluorescent proteins, with 0.65–0.85 in the visible range, surpassing most of the organic dyes (0.3–0.5 in the visible range) [79]. Additionally, surface modifications with different biomolecules helps in targeted bioimaging. This surface conjugation also provides a sensitive analysis of tumour-specific biomarkers, which can benefit cancer diagnosis through the fluorescent property of QDs [80]. This property can also be exploited for live drug pathway tracking [81]. For example, antibody-labelled QDs were found to be suitable for the detection of prostate cancer by targeting the prostate-specific membrane antigen (PSMA) [82]. Similarly, CdSSe/ZnS QDs decorated with anti-estrogen (ER) alpha antibodies were used for the molecular detection of the ER alpha antigen in breast cancer [83]. There are many more such research studies proving that QDs conjugated with different ligands together can be useful in multimodal imaging techniques, particularly in improving cancer diagnosis.

Beyond their role in bioimaging, they generate reactive oxygen species (ROS) through a Type I photoreaction. When a QD absorbs a photon, it creates an exciton, leading to charge separation. Nearby oxygen molecules capture the trapped electron, forming superoxide anions (O_2_^−^). In aqueous conditions, superoxide may react with water or be converted by enzymes like superoxide dismutase into hydrogen peroxide (H_2_O_2_). This process effectively converts light energy (visible light) into ROS production, which was found to be highly potent for cancer cell death in nasopharyngeal carcinomas [84], breast cancer [85], lung cancer [86], and neuroblastoma [87]. Studies have shown that, quantitatively, higher doses of QDs generate maximum ROS [86]. Furthermore, QDs greatly help improve fluorescence resonance energy transfer (FRET)-based assays by being efficient energy donors, thereby enhancing luminescence and playing a pivotal role in photodynamic therapy [88]. SQDs (CdTe, CdSe) have been reported to show the sustained strong absorption and long emission wavelengths required for photodynamic therapies [89]. It has also been observed that surface functionalisation levels up the ROS production; for example, oxygen functional groups on GQDs’ surface helped generate greater amounts of ROS after irradiation [90]. Through nitrogen functionalisation, GQDs were able to produce a greater amount of ROS than nitrogen-free GQDs [91].

In addition to this, a beneficial feature of QDs is their potential to deliver drugs to cancerous tissues efficiently [92] by bypassing biological barriers [93]. Researchers have shown that conjugating drugs onto QDs offers a notable improvement in drug delivery to cancer cells when compared to conventional drug carriers. This targeted approach not only minimises systemic toxicity but also improves the treatment efficacy through targeted drug delivery [81].

**Figure 4:**
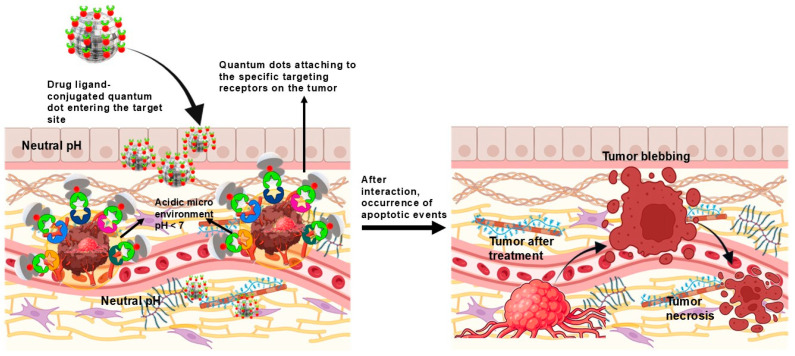
This schematic illustrates the targeted action of drug-ligand-conjugated QDs in delivering chemotherapeutic drugs to varied cancers that bind to specific receptors on tumour cells in an acidic tumour microenvironment (pH < 7). Upon entering the tumour site, QDs release the drugs, leading to enhanced tumour treatment outcomes, as shown by tumour blebbing and necrosis post-treatment.

While their applications remain predominantly in preclinical stages, their promising results indicate potential clinical applications in addressing unique challenges across a spectrum of cancers. Over the years, QDs have been functionalised with targeting ligands for an even better-targeted effect, such as antibodies and/or peptides, to specifically bind to cancer cells, enabling enhanced accumulation and an elevated uptake of the drug in tumour tissues, as depicted in Figure 4 [94].

### 3.1. Graphene Quantum Dots

#### 3.1.1. Breast Cancer

Research on GQDs has shown significant promise in addressing blood cancer. For instance, GQDs incorporated into carboxymethyl cellulose (CMC)-based hydrogel films delivered DOX for breast cancer. The hydrogels exhibited pH-sensitive behaviour, with carboxyl groups ionising under acidic conditions, enhancing positive charge interactions with cancer cell membranes and improving cellular uptake. Additionally, GQDs improved the hydrogel’s thermal stability [95].

GQDs coupled with titanate nanoflowers (TN) decorated with anti-HER-2 antibodies for fluorescent targeted delivery of DOX presented a new approach to breast cancer therapy. Due to its layered structure, the nanoflowers offered a high specific surface area for drug loading. The nanoconjugate not only enhanced the targeted delivery of drugs to HER2-positive breast cancer cells, but also significantly boosted cytotoxicity and cellular uptake. Nanoflowers were covalently linked to GQDs, which aided in the fluorescent tracking of the nanocarriers. The controlled release of the drug from these advanced nanocarriers makes them far more effective than free DOX. Compared to free DOX, the IC50 of DOX-GQDs was significantly lower, making it three times more effective. Moreover, the labelled drug-loaded nanocarriers exhibited an even lower IC50 value, highlighting the enhanced targeting efficiency of anti-HER2 labelling [96].

In another relevant research study, nitrogen-doped blue fluorescent GQDs loaded with methotrexate (MTX) displayed a time-dependent cytotoxicity, with sustained drug release resulting in greater cell death compared to free MTX. The study confirmed the biocompatibility and non-toxic nature of the GQDs, while the sustained drug release provided enhanced therapeutic outcomes [97].

Similarly, GQDs/methylene blue (MB) with DOX were encapsulated in poly lactic-co-glycolic acid (PLGA) nanoparticles enveloped with a coating of bovine serum albumin (BSA). The nanocomplex offered superior antitumour efficacy, combining photothermal therapy and drug delivery under NIR rather than chemotherapy alone. The MB DOX/PLGA nanoparticles generated reactive oxygen species (ROS) under laser irradiation, which led to efficient tumour cell destruction. Further studies on long-term toxicity and biodegradability will provide deeper insights into their clinical applicability [98].

Continuing the pursuit of targeted therapy, the use of a MgAl-layered double hydroxide (LDH) modified polyaniline (PAN) N-GQD nanocomposite loaded with DOX for targeted delivery against MCF-7 cells. The system demonstrated controlled drug release, with a pH-sensitive release under acidic conditions. The unloaded nanocarrier was non-toxic against normal cells at concentrations as high as 100 µg/mL. Contrastingly, the drug-loaded nanocarrier at the same concentration reduced the cell viability to 60%. The hemolysis rates of drug-loaded N-GQDs were elevated as compared to unloaded N-GQDs. However, it is necessary to determine the efficacy of the nanocarrier at preclinical and clinical levels [99].

Another research study used glucosamine (Gl)-conjugated GQDs loaded with curcumin (CUR) to target GLUT receptors overexpressed on breast cancer cells. The pH-sensitive release profile of the Gl-CUR-GQDs led to enhanced cytotoxicity against MCF-7 cells, improving the targeting efficacy and drug delivery compared to unconjugated QDs. However, determining the toxicity levels of Gl GQDs is necessary for a clearer understanding [100].

#### 3.1.2. Colorectal Cancer

GQDs with zeolitic imidazolate framework-8 (ZIF-8), functionalised with PEG and epithelial cell adhesion molecule (EpCAM), were developed and targeted against colorectal cancer. The nanocarrier facilitated the pH-responsive, sustained release of DOX in acidic conditions. Combined with radiotherapy (3 Gy), the nanoconjugates significantly reduced tumour volume and HT-29 colonies in mice. PEGylation improved hematocompatibility, demonstrating its potential for clinical applications [101].

#### 3.1.3. Lung Cancer

Gefitinib-conjugated polyethylene glycol (PEG)-passivated GQDs were incorporated into poly-lactic acid (PLA) microspheres for targeted therapy for lung cancer. The nanocomplex exhibited an acidic pH-sensitive biphasic release pattern. The microspheres showcased enhanced anticancer efficacy against NCI-H522 lung cancer cells, significantly better than free gefitinib. The treated cells exhibited clear signs of early or late apoptosis, with visible nuclear shrinkage and membrane damage. Further, in vivo studies are required to validate the clinical potential and safety of the drug delivery system [102].

Fe_3_O_4_-CTD-GQDs microspheres integrating magnetic carbon, functionalised with 3-aminopropyltrimethoxysilane, and a third-generation triazine dendrimer, covalently conjugated to GQDs to deliver DOX, were developed in another study. The nanoconjugate ensured targeted drug delivery in acidic tumour environments while minimising premature release at physiological pH. They exhibited a dose-dependent reduction in the viability of A549 lung cancer cells with elevated cytotoxicity and greater intracellular retention. Further, in vivo investigations are needed to establish therapeutic efficacy and safety [103].

#### 3.1.4. Pancreatic Cancer

Human serum albumin (HSA)-conjugated GQDs loaded with gemcitabine (Gem) exhibited a biphasic drug release pattern, combining an initial burst with sustained release. The nanocarrier demonstrated selective internalisation and reduced cell viability in Panc-1 cells compared to the free drug, with minimal toxicity to healthy cells. Further in vivo studies are needed [104].

HA (hyaluronic acid) functionalised Ag-GQDs nanocomposite loaded with 5-FU HSA nanoparticles that targeted CD44 receptors on cancer cells, enhancing uptake by Panc-1 cells. HSA and HA–HSA nanoparticles were quite biocompatible and showed almost no toxicity to Panc-1 cells. These nanoparticles displayed excellent biocompatibility with healthy cells, showing minimal toxicity even at high concentrations [105].

Further research investigated the targeted co-delivery of DOX and curcumin (CUR) GQDs for the treatment of breast cancer, wherein the MiGRD peptide for targeting αv integrin receptors on cancer cells significantly boosted cellular uptake. The DOX/CUR complexes had a more significant tendency to accumulate at tumour sites when compared to free drugs. Further pharmacokinetic and toxicity studies would provide improved insights into co-delivery by QDs for breast cancer therapy [106].

Overall, GQDs offer multiple advantages. They have been minimally toxic, indicating their biocompatibility. Their inherent fluorescence property is useful in photothermal therapy, along with their ability to amplify therapeutic efficacy (e.g., there is a threefold increase in the potency of DOX titanate nanoflowers). Functionalised GQDs (e.g., with glucosamine or hyaluronic acid) selectively targeted the overexpressed receptors. Their conjugation with multiple ligands and drugs indicated their versatility for surface conjugation.

#### 3.1.5. Blood Cancer

In another study, imatinib was functionalised with GQDs to act against multiple myeloma cells. While imatinib alone was strongly cytotoxic, imatinib GQDs displayed intermediate cytotoxicity, suggesting a diminished efficacy in comparison to free imatinib, likely attributable to delayed drug release from the GQDs. Similarly, imatinib markedly increased the quantity of apoptotic cells, whereas imatinib GQDs resulted in minimal cell death. This indicated that conjugating drugs to GQDs impeded the former’s rapid action. However, in vitro drug release and in vivo studies are necessary to enhance their effectiveness [107]. While GQDs amplify drug uptake in multiple myeloma cells, their controlled release profile warrants further refinement for enhanced therapeutic outcomes.

#### 3.1.6. Cervical Cancer

Mesoporous silica nanoparticles (MSNs) coated with N-GQDs have been functionalised with HA to target CD44 receptors. N-GQDs with high quantum yield provided brighter cell imaging, and HA-functionalisation demonstrated improved intracellular uptake with sustained fluorescence and stability over months. This nanocarrier enabled prolonged imaging and targeted drug delivery, confirmed by intracellular localisation through fluorescence imaging. Cytotoxicity assays provided limited information, stating that the survival rate of HeLa cells depended on DOX concentration. In vivo and detailed in vitro studies are required for a clear understanding [108]. Hyaluronic acid (HA)-functionalised GQDs targeted CD44 receptors on cervical cancer cells (HeLa), reducing cell viability dose-dependently while sparing normal cells (L929). This system showed high biocompatibility, warranting further in vivo studies [109].

### 3.2. Carbon Dots

#### 3.2.1. Brain Cancer

Triple-conjugated CDs loaded with temozolomide, transferrin, and epirubicin have shown exceptional efficacy in glioblastoma treatment. This system targets cancer cells via receptor-mediated endocytosis, reducing cell viability in brain tumour cell lines more effectively than dual conjugates or free drug combinations. Notably, the synergistic effects of these conjugations enhanced cytotoxicity by 86%, even at reduced drug doses, underscoring their potential for treating various brain tumour cell lines. Further in vivo validation is needed to confirm their therapeutic efficacy [110].

#### 3.2.2. Breast Cancer

In another study, CDs conjugated with DOX demonstrated high drug-loading capacity and pH-responsive release in the acidic tumour microenvironment, enhancing cellular uptake and cytotoxicity. This system efficiently delivered DOX to the cell nucleus, significantly improving apoptotic effects compared to free DOX [111].

In a noteworthy study, combining CDs with gold nanorods (GNs) to deliver DOX, a unique tri-functional nano-worm system enabled not only effective drug delivery but also bioimaging and photothermal therapy. Unlike conventional drug carriers, this system exhibited rapid NIR-triggered release along with high drug loading of CDs. This synergistic approach significantly increased the cytotoxic effects on MCF-7 cells, making it a promising approach for theranostic applications [112].

#### 3.2.3. Cervical Cancer

In another study, folic acid (FA) and boric acid conjugated CDs encapsulating DOX in β-cyclodextrin were synthesised. While FA provided specific targeting for the overexpressed folate receptors, controlled drug release was fostered by boric acid. The pH-sensitive bond between boric acid and β-cyclodextrin ensured sustained drug release, maintaining activity until reaching the tumour site for enhanced cytotoxicity. In vivo studies are essential to validate efficacy and safety [113].

Wen’s research introduced nitrogen and sulphur co-doped CDs loaded with mitoxantrone (MTO), which emitted bright orange fluorescence for extended imaging. These CDs demonstrated exceptional intracellular targeting and sensitivity to folic acid, enhancing their therapeutic efficacy while enabling long-term real-time monitoring of drug delivery [114].

A pH-responsive delivery system using cubosome-loaded DOX-loaded CDs significantly reduced tumour volumes in H22 tumour-bearing mice while sparing healthy cells. It offered an integrated approach combining targeted drug delivery, real-time diagnostic capabilities, and reduced toxicity, making it an ideal candidate for further development in cancer treatment protocols. However, future studies focusing on long-term safety are essential [115].

#### 3.2.4. Liver Cancer

The CD-PEI-DOX nanocomplex has exhibited pH-sensitive drug release tailored for acidic tumour environments, specifically targeting liver cancer cells while sparing healthy cells. The nanocomplex demonstrated superior tumour growth inhibition and enhanced DOX accumulation in liver cancer cells compared to free DOX. The biodistribution of DOX CDs in the MHCC-97L tumours of mice was much higher than that of the free DOX, indicating upgraded pharmacokinetics of the nanocomplex. Long-term biocompatibility and immunogenicity assessments are required [116].

CDs were conjugated with DOX combined with lipid-coated calcium phosphate (LCP) nanoparticles for treating liver cancer. The nanocomplex showed pH-dependent drug release: at acidic pH, it released DOX in a burst followed by sustained release, while at physiological pH, release slowed due to the LCP shell. DOX from LCP-DOX CDs entered cells more gradually through endocytosis, causing a slower but more effective cytotoxic effect than that of the free DOX, rapidly inhibiting tumour growth. In vivo, LCP-DOX CDs significantly reduced tumour volume with minimal systemic toxicity. While the findings position LCP-DOX CDs as a productive approach for managing liver cancer, a detailed assessment of the long-term safety profile of the nanoconjugate would aid in its clinical implementation [117].

Oxaliplatin conjugated with luminescent CDs significantly enhanced its anticancer efficacy compared to the free drug. In vivo, the nanocomplex was able to lower the tumour volume to 11 times smaller than the original tumour volume. Fluorescence imaging revealed gradual drug distribution, although tumour scabbing led to fluorescence decline. Further studies are needed to evaluate safety and long-term effects [118].

#### 3.2.5. Adenoid Cystic Carcinoma

DOX-loaded CDs were developed for nucleus-targeted therapy against adenoid cystic carcinoma. CDs demonstrated high safety for normal cells, even at high concentrations. However, the CDs-DOX complex exhibited significant antitumour effects at acidic pH. Treated cancer cells showed a notably lower survival rate compared to controls or free DOX. Flow cytometry confirmed higher apoptosis rates with the CDs-DOX complex. Detailed in vivo toxicity studies and therapeutic validation are essential to confirm these findings [119].

#### 3.2.6. Lung Cancer

The effectiveness of siMRP1(siRNA with multi-drug resistant protein)-conjugated DOX-loaded poly-ethylenimine (PEI) modified CDs to mitigate chemoresistance in lung cancer was studied. The nanodrug enhanced DOX uptake, evidenced by increased fluorescence, and effectively inhibited cell proliferation. In xenograft models using A549/ADM cells, tumour volume and weight were markedly reduced. Bio-distribution studies showed that the siMRP1-PEI-DOX CDs formulation was specifically accumulated in tumour tissues more effectively than free DOX [120].

Considering all these research findings, it can be summarised that CDs are biocompatible, facilitate pH-triggered drug release, and can be used for theranostic purposes. Also, nitrogen and sulphur doping has been beneficial for extended imaging. CDs help reduce chemoresistance and promote drug accumulation, specifically in tumour tissues.

### 3.3. Carbon Quantum Dots

#### 3.3.1. Breast Cancer

In researching the effect of QDs on breast cancer, a pyrimidine analogue, 5-fluorouracil (FU), was conjugated onto CQDs, which exhibited minimal toxicity to healthy cells and demonstrated dose-dependent cytotoxicity against MCF-7 cells. Also, the 5-FU CQDs elevated the drug potency while minimally harming the healthy tissues [121].

In another study, curcumin-loaded chitosan CQDs combined with Fe_2_O_3_ showed controlled pH-dependent drug release, with CQDs facilitating drug tracking and demonstrating potent anticancer activity against MCF-7 cells. The Fe_2_O_3_ component provided additional stability and anticancer properties, amplifying the therapeutic potential of this nanocomposite. CQDs facilitated controlled pH-dependent release with significant apoptotic and necrotic activity for MCF-7 cell lines. Further, in vivo studies would provide clarity regarding the toxicity and biocompatibility of CQDs [122].

Similarly, dysprosium-doped CQDs loaded with camptothecin (CPT) showed 100 h of sustained pH-responsive drug release, and the nanocomposite exhibited dose-dependent cytotoxicity. The Dy-CQDs’ superparamagnetic properties could benefit magnetic resonance imaging and targeted therapy. Further, research on in vivo environments would help in toxicity assessments [123].

Along the same line, transferrin (Tf)-conjugated DOX-loaded CQDs were developed to act against breast cancer cells. These smart nanocarriers released more DOX in acidic tumour environments while targeting cancer cells via transferrin receptors. This indicated targeted tumour therapy of Tf-DOX CQDs. Tf-DOX CQDs were twice as cytotoxic as the free DOX. Additionally, fluorescent imaging confirmed high cellular uptake of Tf-DOX CQDs by the MCF-7 cells [124].

Lastly, nitrogen-doped CQDs decorated with quinic acid were synthesised and surface-conjugated with gemcitabine (Gem), forming a multifunctional nanoplatform for simultaneous bioimaging and drug delivery. Their vivid blue luminescence enabled tumour-specific fluorescence imaging, while quinic acid facilitated the selective targeting of angiogenic factors overexpressed in MCF-7 cells. These smart nanocarriers released most of the drug payload in the acidic tumour microenvironment while sparing healthy tissues. Cytotoxicity assays and in vivo imaging confirmed the nanoconjugate’s better therapeutic potential, outperforming free Gem and effectively homing in on tumours. Furthermore, in vivo studies confirmed the preferential accumulation of Gem nanoconjugate, which is evident by fluorescence imaging. The combined imaging and drug delivery properties confirmed its theranostic capability [125].

#### 3.3.2. Cervical Cancer

Magneto-fluorescent DOX-loaded CQDs conjugated with FA were prepared from waste crab shell along with the incorporation of transition metal ions, like Gd3+ (Gadolinium (III) ion), Mn2+ (Manganese (II) ion), or Eu3+ (Europium (III) ion), which produced a magnetic field due to their high electronic spin. These different CQDs, including GdCQDs, MnCQDs, and EuCQDs, effectively inhibited cancer cell growth in HeLa cells while showing minimal cytotoxicity on HeLa, HepG2, and HeLung cells, indicating their biocompatibility. The in vivo biocompatibility in zebrafish embryos demonstrated a good survival rate and hatching rate. In the zebrafish embryos, no mortality was seen, even at the highest dosage tested (1000 μg/mL). Pharmacokinetic studies would provide a better understanding of this system’s safety and efficacy [126].

In brief, CQDs are versatile in enabling live drug tracking and fluorescent bioimaging, which could help in the early diagnosis of cancers, while being biocompatible in vivo, which could be promising for use at the clinical level.

### 3.4. SQDs

#### 3.4.1. Breast Cancer

Methotrexate-loaded AgInS2/ZnS QDs exhibited comparable apoptotic efficacy to free drugs, with improved biocompatibility in HeLa cells. Further studies on stability, pharmacokinetics, and immunogenicity are needed to confirm clinical viability [127].

NIR Ag_2_S QDs conjugated with FA and loaded with DOX targeted folate receptor-positive HeLa cells, which showed double the intracellular uptake compared to receptor-negative cells. They showed enhanced apoptotic effects and reduced cell viability in a concentration-dependent manner, outperforming free DOX [128].

#### 3.4.2. Cervical Cancer

ZnO QDs used as “nanolids” to cap MSNs loaded with DOX offered pH-sensitive drug release in the acidic tumour microenvironment. The nanocomplex demonstrated exceptional efficacy by significantly reducing cancer cell viability, showcasing superior specificity compared to free DOX. The secret to its success lay in the dissolution of ZnO nanolids in the acidic tumour microenvironment. The combination of targeted drug release and inherent antitumour activity positioned this system as a promising platform for drug delivery and cancer therapy. However, additional in vivo and toxicity studies are warranted to confirm its therapeutic efficacy [129].

#### 3.4.3. Lung Cancer

Ag-In-Zn-S QD nanoconjugate tailored with L-cysteine, mercaptoundecanoic acid, and lipoic acid selectively targeted FA receptors. They exhibited potent apoptosis-inducing effects and inhibited colony formation in a concentration-dependent manner, surpassing free DOX. Further, in vivo studies are required to confirm their efficacy [130].

ZnO QDs decorated with adipic dihydrazide and heparin loaded with paclitaxel (PTX) exhibited controlled release, surpassing the efficacy of free PTX. The cell viability of the nanocomplex was higher than that of free PTX, lasting longer than that of the free drug. In vivo pharmacokinetic studies demonstrated higher PTX retention in blood plasma, indicating prolonged therapeutic activity [131].

An FA-targeted β-cyclodextrin QD carrier (Ag-In-Zn-S QDs) was developed (QDgreen-β-CD-FA) for delivering C-2028, an unsymmetrical bisacridine anticancer agent, to target lung (H460) and prostate (Du-145, LNCaP) cancer cell lines. This nanoconjugate enhanced tumour targeting while reducing normal cell toxicity. It effectively induced apoptosis in Du-145 and LNCaP cells. A dramatic decrease in senescence markers in lung cancer cells was attributed to folic acid’s telomere-protective properties. The reduction in cancer cell mobility observed, particularly in prostate cancer, indicated significant inhibition of tumour spread. Further in vivo studies are necessary to validate the results [132].

#### 3.4.4. Oral Carcinoma

MTX-loaded L-cysteine-capped CdSe QDs targeted drug-resistant KB cancer cells through overexpressed folate receptors. MTX QDs exhibited significantly higher cytotoxicity toward KB cells compared to free MTX, with an IC50 8.75 times lower than the latter. The nanoconjugates maintained high colloidal stability in biological fluids while retaining a high quantum yield and small hydrodynamic diameter. However, in vitro and in vivo studies are necessary to prove its efficacy [133].

#### 3.4.5. Skin Cancer

Fe_3_O_4_-Ag_2_O, a nanostructured composite incorporated onto cellulose nanofibers, was utilised as a carrier for Etoposide and MTX, specifically targeting skin cancer. This nanostructure integrates Fe_3_O_4_ for superparamagnetic properties and Ag_2_O, a p-type semiconductor, contributing to potential quantum confinement effects at the nanoscale. The nanocomposite significantly reduced cell viability in a concentration-dependent manner, while antioxidant tests revealed a substantial free radical scavenging capacity. Additionally, its ferromagnetic properties present opportunities for dual therapeutic effects and imaging applications. However, further investigations are required to validate its clinical feasibility and to better understand its physicochemical properties at the nanoscale [134].

Conclusively, it can be stated that Ag QDs could improve the intracellular uptake of drug-conjugated QDs, while ZnO QDs acted as a shell that specifically dissolved in the acidic environments, providing pH-sensitive drug release. Moreover, CdSe QDs were able to retain high quantum yield, and BPQDs have been exceptionally brilliant, serving a dual purpose of photothermal therapy and drug delivery.

### 3.5. Others

#### Osteosarcoma

A novel approach to treating osteosarcoma utilised black phosphorus QDs (BPQDs) loaded with DOX and incorporated in a hybrid membrane derived from osteosarcoma cell membrane (OCM) and platelet cell membranes (OPM). This hybrid membrane enabled immune evasion, targeted cancer cell delivery, and resistance to oxidative degradation, thereby improving stability and circulation time. NIR irradiation triggered a controlled release of DOX, enhancing the treatment’s effectiveness. In vitro, the BPQDs-DOX-OPM showed a potent antitumour effect with a higher apoptosis rate. In vivo, BPQDs-DOX-OPM showed superior tumour growth inhibition compared to other groups, leveraging the synergy between photothermal therapy and chemotherapy. Extensive necrosis and inflammatory cell infiltration were observed, highlighting its therapeutic potential. However, detailed in vivo toxicity studies are essential to further evaluate the safety and biocompatibility of BPQDs [135]. Table 1 describes the key advancements in chemotherapeutic drug delivery.

## 4. Challenges Involved

QDs have emerged as promising candidates for cancer therapy due to their unique optical and electronic properties. However, several challenges must be addressed to fully leverage the benefits of QDs for cancer therapy.

### 4.1. Potential Toxicity

One of the primary concerns in utilising SQDs for cancer therapy is their toxicity. SQDs often contain heavy metal elements such as Cd or Pb, which can pose risks at the cellular level. Their release, in particular, can induce cell damage and trigger inflammatory responses [136]. Research to develop biocompatible QDs using materials like carbon, biomolecules, and silicon is underway, which would overcome the cytotoxic effects of QDs [137]. The safety profile of these alternative materials and the long-term effects of quantum dot exposure in the body require thorough investigation to ensure their biocompatibility and minimise the associated adverse effects [137]. Moreover, the interaction of SQDs with cellular components and signalling pathways can disrupt normal cellular functions, leading to impaired cellular homeostasis, proliferation, and survival [138]. To overcome these toxicity concerns, the surface of the QDs can be functionalised with biocompatible molecules, such as shells like zinc [139] and cysteine [140] or a surface modified by polyethylene glycol (PEG) [141,142] or gelatin [143]. On the other hand, CdTe QDs have been used to target NF-κB signalling pathways to achieve apoptotic effects in mice [144], which may be studied at the clinical level, indicating that Cd QDs can benefit chemotherapies. In contrast, carbon-based QDs and GQDs are minimally toxic [35,55].

### 4.2. Stability and Pharmacokinetics

Another significant challenge is the stability and pharmacokinetics of QDs in a biological environment. QDs can undergo degradation, compromising their performance as drug-delivery vehicles [145]. Strategies such as surface modification [146] and encapsulation techniques are being explored to enhance these parameters [147]. These strategies aim to protect QDs from degradation by biological components, ultimately prolonging their circulation time and enhancing their bioavailability within the body [146,147].

### 4.3. Targeting Specificity

The successful conjugation of ligands to QDs is crucial for targeted delivery to cancer cells. Firstly, QDs often have reactive surfaces that can lead to non-specific binding of ligands, reducing the specificity of drug delivery [148]. Another aspect that complicates the conjugation process is the potential impact on the optical properties of QDs. Conventional conjugation techniques may alter the QDs’ optical characteristics, affecting their efficacy in simultaneous imaging and therapy. Thus, optimised conjugation techniques that preserve QD optical properties while ensuring targeted ligand attachment are needed to overcome this hurdle [66].

### 4.4. Minimal Clinical Information

Despite significant preclinical advancements, research on QDs remains in its early stages, with limited clinical trials. As of December 2024, only six clinical trials for QDs have been registered, with two being subsequently withdrawn, as described in Table 2 [149]. This highlights the need for further translational studies to bridge the gap between preclinical research and clinical application.

## 5. Future Prospects

The versatility of QDs opens avenues for innovative strategies in cancer therapy. Recently, it has been found that black phosphorus material acts as a “nanoknife” along with ROS generation, enabling precise tumour ablation as discussed in the text. BPQDs are being researched in the context of chemotherapeutic drug delivery, and may possibly be used as both nanoknives and nanocarriers [156].

The future requires the use of non-toxic, biocompatible QD materials along with complete information on the related pharmacokinetics and pharmacodynamics to facilitate their near-term translation from research to clinical applications.

QDs with surface modifications can be used for precise gene delivery. Recently, work on QD gene delivery at the cellular level has been reported, but has yet to reach preclinical and clinical levels [157].

Future QD-based therapies may incorporate immunomodulatory functions. QDs functionalised with immune checkpoint inhibitors or stimulatory molecules could enhance immune system recognition and attack cancer cells. Such systems could synergise with chemotherapy and radiation for comprehensive cancer treatment.

## 6. Conclusions

Integrating nanotechnology with pharmaceutical and medical sciences through QDs significantly advances cancer diagnosis and treatment. Their unique optical and physicochemical properties and the ability to deliver drugs in a targeted and controlled manner position QDs as a versatile tool in oncology. This review has highlighted the diverse applications of different types of QDs across various cancers, demonstrating their ability to enhance therapeutic efficacy, minimise systemic toxicity, and enable real-time imaging.

However, substantial challenges remain. The potential toxicity of QDs arising from their heavy metal content raises critical concerns regarding long-term safety. Additionally, issues such as their stability and pharmacokinetics need to be addressed to unlock their clinical potential. The current lack of comprehensive clinical data further emphasises the need for translational research to bridge the gap between preclinical findings and real-world applications.

Nevertheless, ongoing advancements in QD technology are addressing these limitations. Efforts to develop biocompatible and biodegradable QDs, optimise drug conjugation techniques, and enhance stability and targeting specificity are paving the way for clinical translation. Emerging innovations such as QD-based immunomodulation, gene delivery, and precision ablation techniques hold great promise for expanding the scope of QD applications in cancer therapy.

In conclusion, the convergence of nanotechnology and oncology holds immense promise for revolutionising cancer therapy. By enabling personalised, targeted treatments with enhanced efficacy and reduced side effects, QD-based drug delivery systems are poised to transform the landscape of cancer therapy. This review highlights the critical need for interdisciplinary collaboration and sustained research efforts to unlock the full potential of QDs in clinical oncology.

## Figures and Tables

**Table 1:** QDs used for drug delivery in various cancers with their key findings.

S.No.	Type of QDs	Drug	Target Disease	Inference	Reference
1.	Ag QDs	Methotrexate	Cervical cancer	IC50 values of drug-loaded targeted QDs were found to be comparable to those of free drug in cancer cell inhibition. No significant cytotoxicity was observed after 24 h.	[127]
2.	Ag QDs	Doxorubicin	Cervical cancer	2.8 times decrease in IC50 values for FA-DOX QDs as compared to free DOX. 3-fold enhancement in the intracellular uptake due to FA conjugation. 2.5-fold increment in the M30 levels compared to free DOX in HeLa cells.	[128]
3.	Ag QDs	Doxorubicin	Lung Cancer	FA conjugation augmented the cytotoxic action of the DOX-MUA-QDs. FA-MUA-DOX QDs obstructed the colony-forming ability of A549 effectively at concentrations near their cytotoxic thresholds. DNA damage in the cells treated by FA-MUA-DOX QDs was genotoxic to the A549 cells.	[130]
4.	Ag QDs	Etoposide + methotrexate	Skin Cancer	Ferromagnetic QD nanocomposites showed sustained release for 30 h. Reduced cell viability in a concentration-dependent manner.	[134]
5.	Ag-In-Zn QDs	Compound C-2028	Lung and prostate cancer	Strong cytotoxic action against cancer cells with low IC80 values. Cytotoxicity reduced upon conjugation with β-cyclodextrin but vice versa with folic acid conjugation.	[132]
6.	BPQDs	Doxorubicin	Osteosarcoma	Prominent aggregation of DOX in the tumour tissue from the BPQDs. The stability of BPQDs is augmented through OPM encapsulation. NIR light irradiation triggered DOX release. In vivo studies showed notable tumour reduction.	[135]
7.	CdSe QDs	Methotrexate	Throat cancer	Higher cytotoxicity by drug conjugate than free MTX with 4 times more efficacy.	[133]
8.	ZnO QDs	Paclitaxel	Lung Cancer	Acidic pH favoured sustained drug release observed. Cell viability was inversely proportional to drug concentration. PTX nanocomposite had a more pronounced antitumour effect than free PTX. Longer PTX retention time achieved by the nanocarrier in vivo.	[131]
9.	CDs	Doxorubicin	Lung Cancer	siMRP1 (siRNA) improved the permeability inside the nuclei. Effectively downregulated the expression of MRP1, increasing intracellular drug accumulation. The tumours exhibited significantly reduced size and weight compared to those treated with free DOX. CD-DOX-siMRP1 had the lowest IC_50_ value (17.4 µg/mL) than DOX CDs and free DOX.	[120]
10.	CDs	Epirubicin and Temozolomide	Brain Tumour	Triple conjugated system showed profound cytotoxicity in tumour cells with minimal effects on normal cells. Conjugating with transferrin improved the cytotoxicity and cellular uptake of conjugated QDs.	[110]
11.	CDs	Doxorubicin	Breast Cancer	DOX-CDs complex exhibited higher cellular uptake and apoptosis in cancer cells compared to free drugs. Significantly superior inhibition capacity in cancer cells.	[111]
12.	CDs	Doxorubicin	Breast Cancer	Rapid drug release under NIR radiation. Drug release triggered at pH 7.2 was favourable for cancer cells that did not undergo anaerobic glycolysis. Showed low IC50.	[112]
13.	CDs	Doxorubicin	Cervical and kidney cancer	The β-cyclodextrin decorated CDs showed acidic pH-dependent drug release. Selective targeting of cancer cells achieved upon decoration with β-cyclodextrin.	[113]
14.	CDs	Mitoxantrone	Cervical cancer	Concentration-dependent inhibition effect. Energy-dependent cell uptake facilitated by endocytosis.	[114]
15.	CDs	Doxorubicin	Cervical cancer and liver cancer	Showed 3 times higher fluorescence intensity of DOX CDs cubosomes at 24 h. Significantly lower cell viability by DOX CDs cubosomes. In vivo DOX CDs cubosomes exhibited the lowest average tumour volume. Reduced cardiotoxicity and hepatotoxicity of DOX CDs cubosomes.	[115]
16.	CDs	Doxorubicin	Liver cancer	Increased drug release at pH 5.2. Sustained release from CDs (72 h), whereas free DOX released completely in 10 h. DOX-PEI CDs had stronger fluorescence in tumours and improved drug delivery compared to free DOX. The DOX-PEI CDs animal group had better survival than the free DOX group, indicating a superior safety profile for the nanoconjugate.	[116]
17.	CDs	Doxorubicin	Liver cancer	Burst release at acidic pH with sustained release. Higher cytotoxicity exhibited by LCP-DOX CDs compared to free dox, with cell viability dependent on DOX concentrations. Efficient internalisation of LCP-DOX-CDs. The highest tumour growth inhibition rate was achieved by the LCP-DOX CDs after in vivo study.	[117]
18.	CDs	Oxaliplatin	Liver Cancer	Low cytotoxicity suggested good biocompatibility. Significantly reduced the tumour volume in mice within 6 days. Real-time tracking facilitated by CDs. Stable mice body weight indicated low toxicity of nanoconjugate.	[118]
19.	CDs	Doxorubicin	Adenoid Cystic Carcinoma	DOX-CDs biocompatible and non-toxic to normal cells. DOX-CDs-induced apoptosis inhibited tumour growth and prolonged the survival of the mice.	[119]
20.	CQDs	5-fluorouracil	Breast Cancer	The nanoconjugate exhibited higher cytotoxicity than the free drug. Better release profile in acidic environments.	[121]
21.	CQDs	Curcumin	Breast Cancer	Viable cancerous cell count was lowered, and late apoptosis was boosted in MCF-7 breast cancer cell lines upon treatment. Controlled drug release by curcumin CQDs. The nanocomposite showed pH-dependent drug release owing to chitosan’s pH sensitivity.	[122]
22.	CQDs	Camptothecin	Breast Cancer	Sustained drug release was observed for up to 100 h, followed by a rapid release phase. Dose-dependent cytotoxicity observed. pH-responsive drug release observed.	[123]
23	CQDs	Doxorubicin	Breast Cancer	Elevated drug release was observed at acidic pH. Higher apoptotic effect of transferrin (Tf) DOX CQDs than free DOX. DOX Tf CQDs better internalised than free drugs and DOX CQDs Real-time drug release tracking through CQDs.	[124]
24.	CQDs	Gemcitabine	Breast Cancer	Elevated drug release in acidic pH compared to physiological pH. IC50 value of drug-loaded QDs is 2 times less than the free drug. Drug-loaded QDs were localised in the tumour region, sparing the healthy normal cells.	[125]
25.	CQDs	Doxorubicin	Cervical Cancer	The FA-conjugated CQDs inhibited cell growth in HeLa cells more effectively than the free drug at an equivalent concentration. CQDs had no evident impact on the survival and hatching rates of zebrafish embryos and larvae, indicating good biocompatibility.	[126]
26.	CQDs	Curcumin	Cervical cancer	FACQDs and Curcumin-loaded FACQDs showed biocompatibility and safety. Drug-loaded FACQDs selectively targeted and limited the proliferation of HeLa cancer cells whilst sparing normal cells. Zebrafish embryos had normal survival rates and hatching rates after being treated with FACQDs.	[126]
27.	GQDs	Doxorubicin/Curcumin	Colorectal and breast cancer	Enhanced tumour penetration due to MiRGD peptide Better release profile in an acidic environment, indicating its selective targeting. Higher drug accumulation at the tumour site than that of free drugs.	[106]
28.	GQDs	Gefitinib	Lung cancer	Cell viability is inversely proportional to concentrations of drug-conjugated QDs. pH-sensitive behaviour of QDs favoured its release in an acidic environment.	[102]
29.	GQDs	Doxorubicin	Blood Cancer	Efficient drug release in acidic environments of the cancer cells. GQDs improved the mechanical and thermal stability of the hydrogel matrix.	[95]
30.	GQDs	Imatinib	Blood Cancer	Higher IC50 values were observed for drug-loaded GQDs. Drug-loaded GQDs induced mild apoptosis.	[107]
31.	GQDs	Doxorubicin	Breast Cancer	A high specific surface area is provided by titanate nanoflowers (TN) for drug loading. Enhanced targeting specificity by Anti-HER2 labelled DOX-loaded GQD-TNs. Negligible cytotoxicity was observed for drug-free nanocarriers.	[96]
32.	GQDs	Methotrexate	Breast Cancer	Nitrogen doping offered a larger surface area, resulting in higher drug loading. Sustained release of drug obtained for 48 h under neutral conditions. Dose-dependent cytotoxicity was observed after 48 h.	[97]
33.	GQDs	Doxorubicin	Breast Cancer	Acidic pH favoured drug release. Significant oxygen production efficiency of methylene blue (MB) DOX GQDs. pH-responsive internalisation of MB DOX GQDs in HeLa cells Improved cell viability with MB DOX GQDs upon laser irradiation.	[98]
34.	GQDS	Doxorubicin	Breast Cancer	pH-triggered DOX release. Amplified hemolytic rate with gradual increment in DOX nanoconjugate concentration. Inversely proportional viability to DOX concentration.	[99]
35.	GQDs	Curcumin	Breast Cancer	Glucosamine (Gl)-conjugated curcumin-loaded GQDs upgraded the cytotoxic effects compared to free drugs. Elevated intracellular uptake owing to Gl targeting to glucose transporters.	[100]
36.	GQDs	Curcumin	Cervical cancer	70% of drugs released within 12 h at pH 5 Reduced cell viability. Improved targeting of drug-loaded GQDs by hyaluronic acid	[109]
37.	GQDs	Doxorubicin	Colorectal cancer	EpCAM aptamer conjugated nanoparticles encapsulated DOX, ZIF-8, and GQDs facilitated triple delivery for the treatment of colorectal cancer. Drug release from the nanoconjugate was favoured at acidic pH 5.4 with a burst release prior to gradual release.	[101]
38.	GQDs	Gemcitabine	Pancreatic cancer	Conjugation of GQDs with human serum albumin nanoparticles demonstrated sustained drug release and improved stability. HA targeted CD44 receptors for enhanced delivery to resistant cells.	[104]
39.	GQDs	5-fluorouracil	Pancreatic cancer	Ha-functionalised formulation achieved higher cell inhibition than non-functionalised nanocomposite. HA, conjugation brought down IC_50_ value to half of free 5-FU. Viable cells were reduced upon being treated with Ha-functionalised nanoconjugates.	[105]
40.	GQDs	Doxorubicin	Lung cancer	Dendrimer functionalised drug-loaded QDs showcased a dose-dependent decrease in cell viability with increasing concentrations. Drug-loaded nanoconjugate displayed pH-dependent release.	[103]

Abbreviations: Ag QDs—Silver quantum dots, Ag-In-Zn QDs—Silver-Indium-Zinc quantum dots, BPQDs—Black phosphorus quantum dots, CdSe QDs—Cadmium selenide, CDs—Carbon dots, CQDs—Carbon quantum dots, GQDs—Graphene quantum dots.

**Table 2:** Clinical trials involving quantum dots.

S.No.	Study Title	Purpose	Study Design	Status	Trial Number	Reference
1.	Intravitreal quantum dots (QD) for advanced retinitis pigmentosa (RP) (QUANTUM)	Retinitis Pigmentosa	Randomised Parallel assignment Double masking (participant, outcomes assessor)	Active	NCT05841862	[150]
2.	Clinical trails of photoelectrochemical immunosensor for early diagnosis of acute myocardial infarction	Acute myocardial infarction	Randomised controlled Quadruple masking (participant, care provider, investigator, outcomes assessor)	Unknown	NCT04390490	[151]
3.	Topical fluorescent nanoparticles conjugated somatostatin analogue for suppression and bioimaging breast cancer	Breast cancer	Randomised Parallel assignment Single masking (participant)	Unknown	NCT04138342	[152]
4.	Diabetes autoimmunity withdrawn in new onset and in established patients	Diabetes mellitus, Type 1	Randomised Parallel assignment Triple masking (participant, care provider, investigator)	Active	NCT03895437	[153]
5.	Diabetes autoimmunity withdrawn in new-onset patients (DAWN)	Diabetes mellitus, Type 1	Prospective, randomised Parallel assignment Triple masking (participant, care provider, investigator)	Withdrawn	NCT03794973	[154]
6.	Diabetes autoimmunitY withdrawn in established patients (DAY)	Diabetes mellitus, Type 1	Prospective, randomised Parallel assignment Triple masking (participant, care provider, investigator)	Withdrawn	NCT03794960	[155]
